# Mechanistic Contributions of lncRNAs to Cellular Signaling Pathways Crucial to the Lifecycle of Human Papillomaviruses

**DOI:** 10.3390/v14112439

**Published:** 2022-11-03

**Authors:** Warda Arman, Karl Munger

**Affiliations:** 1 Department of Developmental, Molecular and Chemical Biology, Tufts University School of Medicine, Boston, MA 02111, USA; 2 Molecular Microbiology Program, Graduate School of Biomedical Sciences, Tufts University School of Medicine, Boston, MA 02111, USA

**Keywords:** human papillomaviruses, viral lifecycle, long noncoding RNA, epithelial differentiation, basal cell identity, cellular proliferation

## Abstract

Papillomaviruses are ubiquitous epitheliotropic viruses with double-stranded circular DNA genomes of approximately 8000 base pairs. The viral life cycle is somewhat unusual in that these viruses can establish persistent infections in the mitotically active basal epithelial cells that they initially infect. High-level viral genome replication (“genome amplification”), the expression of capsid proteins, and the formation of infectious progeny are restricted to terminally differentiated cells where genomes are synthesized at replication factories at sites of double-strand DNA breaks. To establish persistent infections, papillomaviruses need to retain the basal cell identity of the initially infected cells and restrain and delay their epithelial differentiation program. To enable high-level viral genome replication, papillomaviruses also need to hold the inherently growth-arrested terminally differentiated cells in a replication-competent state. To provide ample sites for viral genome synthesis, they target the DNA damage and repair machinery. Studies focusing on delineating cellular factors that are targeted by papillomaviruses may aid the development of antivirals. Whilst most of the current research efforts focus on protein targets, the majority of the human transcriptome consists of noncoding RNAs. This review focuses on one specific class of noncoding RNAs, long noncoding RNAs (lncRNAs), and summarizes work on lncRNAs that may regulate the cellular processes that are subverted by papillomavirus to enable persistent infections and progeny synthesis.

## 1. Introduction

Human papillomaviruses are a large family of double-stranded DNA viruses that fall into several phylogenetic genera. The high-risk alpha genus HPVs infect the mucosal epithelia of the oral and anogenital tract and contribute to approximately 5% of all human cancers [1]. These include virtually all cervical carcinomas and a large fraction of other cancers of the anogenital tract, as well as a growing number of oral cancers, particularly oropharyngeal carcinomas.

**Figure 1:**
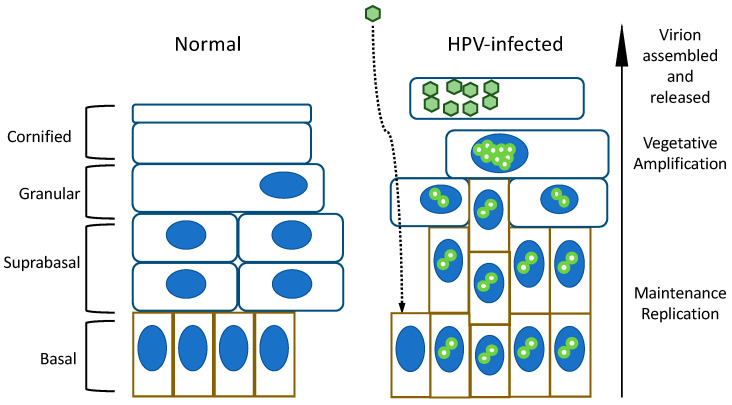
Comparison of normal stratified squamous and an HPV-infected epithelium. Infection occurs in the basal layer that contains proliferative cells. After an initial phase of genome replication in the infected host cells’ nuclei (blue ovals), the viral genomes (green circles) are maintained at a low copy number. Vegetative amplification occurs only in the differentiated layers of the epithelium. High-level viral DNA synthesis takes place in replication factories that form at sites of double-strand host cell DNA breaks. Viral progeny is produced and shed in the denucleated dead skin cells (“squames”) that slough off. See text for details and references.

The HPV lifecycle is linked to the host epithelial cells’ differentiation program [2]. HPVs infect the proliferative cells in the basal compartment of the epithelium, where they initially replicate their genomes at a low copy number. As these cells divide, HPV genomes are partitioned during mitosis to daughter cells and replicate in concert with cellular DNA synthesis [3]. HPV genomes can be maintained in basal cells for decades, and this form of replication is referred to as maintenance replication. When infected basal cells are pushed upwards, they undergo differentiation and stop dividing. High-level viral genome synthesis, the expression of late viral proteins, and the production of viral progeny are restricted to terminally differentiated cells. This form of viral genome synthesis is referred to as “genome amplification” or “vegetative amplification” [4]. Terminally differentiated cells do not express cellular replication factors that HPVs depend on to synthesize genomes, and HPV genomes are replicated at the sites of double-strand DNA breaks [5,6]. Hence, the peculiar replication strategy of HPVs depends on maintaining the basal cell identity of persistently infected cells, inhibiting the differentiation process by keeping differentiating cells in a replication-competent state and affecting DNA double-strand break repair for the formation of HPV replication factories at the sites of DNA damage (Figure 1).

The ability of the HPV E6 and E7 proteins to subvert the normal differentiation program of squamous epithelia has been well-documented (reviewed in [7,8]). For example, HPV16-positive cells will still undergo stratification and some differentiation in organotypic raft culture, but they fail to properly differentiate and continue to express proliferation markers [9]. Consistent with this, the transcriptome changes observed in differentiated HPV16-positive cells, compared with their controls, indicate that keratinocyte differentiation and epithelial barrier function are disrupted by HPV infection [10]. One important mechanism by which high-risk HPV E7 proteins delay epithelial differentiation is to trigger the proteasomal degradation of PTPN14. PTPN14 depletion or deletion caused decreased expression of keratinocyte differentiation markers, including keratins 1, 4, 10, and 16, involucrin, and desmocolin 1, while the expression of the MAF and MAFB transcription factors that are known to regulate epithelial differentiation was markedly decreased [11]. Studies of sophisticated organotypic raft cultures have revealed that HPV16 E7-mediated PTPN14 degradation contributes to the maintenance of basal cell identity [12], which enables long-term, persistent HPV infection of squamous epithelia.

Similarly, E6 and E7 have been shown to engage DNA damage repair (DDR) pathways and increase the incidence of double-strand DNA breaks [13]. Additionally, the components of the DDR pathways are expressed at higher levels in proliferative [4,14] and differentiated HPV-expressing cells [4]. However, the molecular mechanisms that account for these activities have not been fully delineated.

Most studies that aimed at delineating the molecular mechanism by which E6 and E7 affect cellular differentiation and engage DDR signaling have focused on protein targets. With the recent advancements in sequencing technologies, there has been increased annotation of long noncoding RNAs (lncRNAs). LncRNAs are transcripts that are over 200 nucleotides, with a coding potential of less than 100 amino acids [15]. Due to the lack of relevant protein-coding information, the sequences of lncRNAs are typically poorly conserved. Only 10% of human lncRNAs are present as single orthologs in mice [16]. Similar to mRNAs, lncRNAs show discrete tissue- or cell-type-specific expression [17]. 

There is a growing realization that lncRNAs play important regulatory roles in almost every cellular process. Hence, it is not surprising that their expression is dysregulated in many diseases, including cancer [18,19,20], as well as in response to viral infections [21,22,23]. LncRNAs can function by interacting with DNA, RNA, and/or proteins. This has complicated the delineation of their biological activities and the identification of relevant cellular targets. Similar to microRNAs, the activities of lncRNAs can be modulated by small, nucleic acid-based inhibitors and mimics [24], suggesting that they can be targeted for therapy.

This review focuses on how lncRNAs contribute to the regulation of epithelial cell differentiation, how they may contribute to maintaining basal cell identity, and how they modulate DDR signaling.

## 2. Regulation of Epithelial Cell Differentiation

### 2.1. TINCR and DANCR

**Figure 2:**
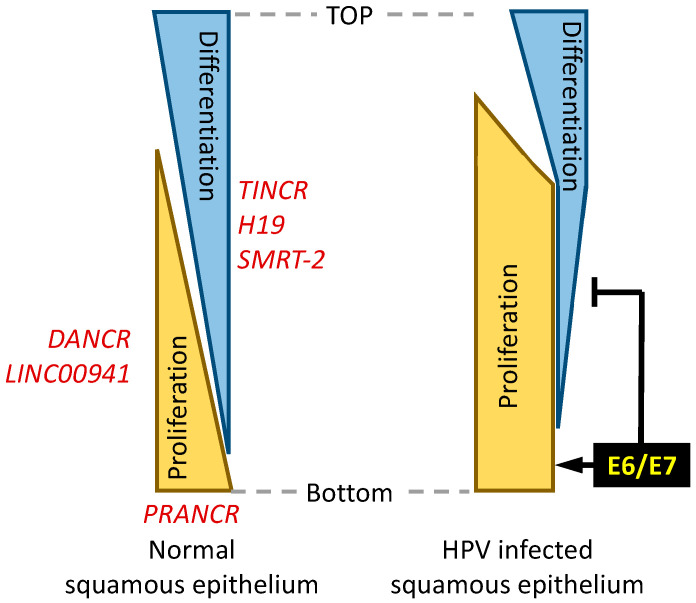
Proliferation and differentiation of normal (**left**) and HPV-infected (**right**) squamous epithelia. In normal squamous epithelia, the processes of proliferation and differentiation are coupled, whereas in HPV-infected epithelia, these processes are uncoupled due to HPV E6 and E7 expression. Consequently, there are an expansion of proliferating, poorly differentiated cells. LncRNAs (shown in red) that inhibit differentiation are shown on the left, while those that drive differentiation are shown on the right. PRANCR has been implicated as a regulator of differentiation and proliferation and is shown at the bottom. See text for details and references.

Two lncRNAs that have been identified as regulators of keratinocyte differentiation are the differentiation antagonist noncoding RNA (DANCR; also termed “anti-differentiation noncoding RNA”(ANCR)) and the terminal differentiation-induced noncoding RNA (TINCR). DANCR and TINCR differentially regulate keratinocyte differentiation; DANCR suppresses whereas TINCR stimulates differentiation. Our RNA sequencing studies have shown that DANCR levels were increased, whereas TINCR expression was reduced in HPV16 E6/E7 expressing human foreskin keratinocytes [25,26] (Figure 2).

DANCR was identified in a 2012 study that analyzed lncRNA expression by next-generation RNA sequencing in epidermal progenitor populations versus differentiating keratinocytes [27]. The authors showed that DANCR expression was downregulated during differentiation. DANCR depletion induced the expression of filaggrin, loricrin, keratin 1, and involucrin and interfered with the expression of the GRHL3 and HOPX transcription factors that regulate epithelial differentiation [28,29]. High-level DANCR expression has been observed in cervical cancer tissues and derived cell lines (HeLa, SiHa, C-33A, ME-180) and correlates with lower overall survival rates in cervical cancer patients [30]. DANCR depletion was shown to inhibit the proliferation of cervical cancer cell lines and resulted in decreased tumor size in mouse xenograft models [30].

TINCR, on the other hand, activated epidermal differentiation, as evidenced by the induction of keratinocyte differentiation markers [31]. TINCR depletion resulted in decreased protein levels of filaggrin, loricrin as well as keratins 1 and 10. Using RNA-interactome sequencing, which involves pulling down endogenous TINCR RNA, they were able to identify TINCR-binding mRNAs. When TINCR expression was ablated, it resulted in the downregulation of these TINCR-binding mRNAs, indicating that TINCR has a role in stabilizing these mRNAs. They also analyzed the protein interactome of TINCR and identified Staufen double-stranded RNA binding protein 1 (STAU1) as a TINCR binding protein. Staufen and related double-stranded RNA (dsRNA)-binding proteins are involved in transporting mRNAs to different subcellular compartments and/or organelles. The depletion of Staufen impairs epithelial differentiation. Hence, TINCR may form a complex with Staufen to stabilize differentiation-associated mRNAs [31].

It is important to note that the lncRNA TINCR is not strictly a noncoding RNA, since it encodes a small peptide that has been detected by mass-spectrometry-based approaches in cornified epithelial cells [32]. It is unclear, however, whether and/or how this TINCR-encoded micropeptide contributes to TINCR’s ability to induce differentiation. However, if noncoding RNAs can encode small functional micropeptides, it suggests that some protein-coding RNAs may also have biological activities akin to those of lncRNAs.

Follow-up studies showed that DANCR and TINCR are essential upstream regulators of the MAF:MAFB transcription factors that are induced in epidermal differentiation both at the mRNA and the protein level [33]. There is some suggestion that MAFB expression may occur to some extent by aiding the binding of EZH2-repressive complexes to H3K27me3. TINCR upregulation of MAF:MAFB also may occur, at least in part, via the major known mechanism of TINCR action, specifically mRNA binding and stabilization [33].

These studies illustrate the power of detailed, rigorous multi-omic approaches in uncovering the molecular mechanisms of action of specific lncRNAs. Unfortunately, many of the published papers on lncRNAs are limited in reporting on their modulation of expression and correlating their expression to biological processes, and thus, they do not provide definitive insights into their mechanisms of action.

### 2.2. Additional lncRNAs That Have Been Implicated in Modulating Differentiation

The following is a short overview of other lncRNAs that have been mechanistically implicated in keratinocyte differentiation.

H19 is one of the best-studied lncRNAs. In primary human keratinocytes, lncRNA-H19 may act as a sponge for miR-130b-3p, which prevents the miRNA from acting on desmoglein 1 (DSG1) [34], a gene that promotes differentiation by suppressing MAPK/ERK signaling [35,36]. In this example, the miRNA inhibits differentiation, while the lncRNA promotes differentiation. Interestingly, RNA seq studies in our group documented an increase in H19 expression in HPV16 E6/E7 expressing keratinocytes [25,26].

PRANCR (progenitor renewal associated with noncoding RNA) has been reported to regulate keratinocyte proliferation, cell cycle progression, and clonogenicity. The downregulation of PRANCR results in increased levels of p21^CIP1^ and increased localization of p21^CIP1^ to the nucleus and affects epidermal tissue homeostasis by regulating proliferation, as evidenced by a decrease in Ki67-positive cells in the basal epidermal strata [37]. However, PRANCR depletion also inhibited differentiation, and the expression of differentiation markers was also reduced upon PRANCR depletion [38]. Interestingly, however, PRANCR was required for epithelial stratification in organotypic “raft” cultures [37]. A recent study suggested that PRANCR can regulate splicing. A total of 238 PRANCR-regulated exonic splicing events were identified, including the fibronectin-1 gene [38]. The authors of this study presented a model whereby PRANCR regulates epithelial differentiation, at least in part, by controlling the relative abundance of the extra domain A (EDA) containing fibronectin isoform, which promotes cell proliferation.

Expression of the SMRT-2 lncRNA is induced during differentiation and knockdown results in reduced expression of multiple differentiation genes (keratin 1, loricrin, filaggrin, and transglutaminase 1). Interestingly, the expression of most genes dysregulated in response to SMRT-2 loss is controlled by the CEBP, KLF4, and ZNF750 transcription factors, which are downregulated upon SMRT-2 depletion. Hence, SMRT-2 may affect the expression of epithelial differentiation proteins by regulating these transcription factors. SMRT-2 is a highly conserved lncRNA that has broad tissue expression, and its expression is reduced in squamous cell carcinomas [39].

LINC00941was identified as a transcript that is enriched in progenitor keratinocytes. It inhibits keratinocyte differentiation by reducing the expression of the small proline-rich protein 5 (SPRR5), a predicted protein within the epidermal differentiation complex, which is expressed during the late stage of epithelial differentiation. SPRR5 depletion results in decreased expression of both early and late differentiation markers. Hence, LINC00941 may regulate keratinocyte differentiation partially by modulating SPRR5 abundance [40]. LINC00941 expression is upregulated in colorectal cancers and is associated with poor prognosis in patients [41]. In this context, LINC0941 has protumorigenic activities. It activates EMT by binding SMAD4, thereby preventing its degradation. This results in the deregulation of the TGF-β/SMAD2/3 axis. This finding may bear relevance to squamous epithelial cells since the TGF-β/SMAD axis importantly regulates keratinocyte proliferation and differentiation [42].

From the studies described in this section, it is clear that a growing number of lncRNAs are implicated in regulating epithelial differentiation (Figure 2). Given the importance of disrupting the link between epithelial differentiation and proliferation for the papillomavirus life cycle, it will be interesting to determine whether some of these lncRNAs are targeted by papillomavirus proteins.

## 3. LncRNAs That Affect WNT/β-Catenin Signaling

The WNT/β-catenin signaling circuit has been implicated in epithelial stem cell maintenance [43], which may be critical for the ability of HPVs to establish and maintain persistent infections of basal epithelial cells. The WNT/β-catenin pathway is aberrantly activated by mutation in many human cancers [44]. Despite exhibiting hallmarks of aberrant WNT signaling such as increased β-catenin staining [45], WNT pathway mutations are not commonly observed in HPV-positive cancers [46,47]. Studies with transgenic mice showed that aberrant WNT signaling can accelerate cervical carcinogenesis. Overall, 94% of HPV16 E7 transgenic mice that also expressed a constitutively active β-catenin developed invasive cervical cancers as early as seven months of age. In contrast, only 50% of HPV16 E7 expressing control mice developed tumors [48]. HPV16 E6/E7 expression causes the activation of WNT signaling, and E6, specifically, was shown to significantly enhance the WNT/β-catenin/TCF-dependent signaling response in a dose-dependent manner [49]. In HPV16-positive oropharyngeal squamous carcinoma cells, E6 and E7 were both shown to trigger the nuclear accumulation of β-catenin and activation of WNT signaling. For a more detailed review of the regulation of the WNT/β-catenin signaling pathway by HPV E6 and E7, see [50]. Given that the WNT signaling pathway plays an important role in oncogenesis, differentiation, and proliferation, we will discuss lncRNAs that play a role in this pathway in some more detail.

We discussed the role of DANCR in regulating proliferation and differentiation in the previous section; however, there is also evidence that DANCR may activate the WNT/β-catenin signaling pathway by interacting with the β-catenin (CTNNB1) mRNA [51,52]. These studies suggest the exciting possibility that DANCR may also affect stem cell properties and/or basal cell identity in HPV-expressing epithelial cells by regulating WNT signaling.

The lncRNA CCAT-1 has also been identified as an effector of WNT signaling. CCAT-1 was found to be upregulated in cervical cancer tissues, compared with adjacent normal tissues, and high expression levels were found to be related to the stage and size of the tumor and recurrence prognosis [53]. CCAT-1 also promoted proliferation and inhibited apoptosis in the Hela and CaSki cell lines, and it was suggested that this is due to the activation of the WNT/β-catenin pathway [53].

It has been previously reported that HOTAIR was downregulated in the HPV16-positive SiHa cervical carcinoma line but was overexpressed in HPV18-positive HeLa cells [54]. HOTAIR has been shown to regulate a variety of gene expression programs through association with epigenetic regulatory complexes, including the polycomb repressive complex 2 (PRC2) [55]. However, at least in HeLa cells, HOTAIR may regulate WNT signaling by downregulating the negative regulators of this pathway such as SOX17 [54]. We showed that HOTAIR expression was lower in HPV16 E6- and E7-expressing human foreskin keratinocytes, compared with control cells [26]. HOTAIR expression is also lower in many cervical lesions and cancers [56,57], although a more detailed analysis revealed that there may be two distinct groups of cervical cancer cases, those that express high HOTAIR and those that express low HOTAIR. High and low HOTAIR-expressing cervical cancers showed differences in gene expression programs that may be relevant to metastasis [58]. HPV16 E7 was shown to bind HOTAIR by RNA immunoprecipitation (RIP), suggesting a model whereby E7 may not only modulate HOTAIR expression but also HOTAIR function [58].

The lncRNA HNRNPU-AS1 has been previously reported as inhibiting the WNT/β-catenin signaling pathway via the miR-205-5p/AXIN2 axis. HNRNPU-AS1 levels were found to be decreased in cervical carcinoma tissue versus adjacent noncarcinoma samples, and low-level HNRNPU-AS1 expression is associated with poor prognosis [59]. HNRNPU-AS1 levels were found to be low in cervical cancer cell lines, most prominently in the HPV-positive cell lines. As expected, the expression of the main HNRNPU-AS1 target, miR-205-5p, was increased as a consequence of low HNRNPU-AS1 expression, whereas AXIN2, an inhibitor of WNT/ β-catenin signaling and the direct target of miR-205-5p, was decreased in these cells. This may explain the finding that WNT/β-catenin signaling may be dysregulated in cervical carcinoma despite the apparent absence of WNT/β-catenin pathway mutations [45,46].

Lastly, the lncRNA SPINT1-AS1 was identified as a cervical-cancer-associated lncRNA. SPINT1-AS1 expression was increased in cervical cancers and correlated with advanced stage and poor prognosis. SPINT1-AS1 was found to repress miR-214 biogenesis, which resulted in the upregulation of β-catenin, a target of miR-214. The expression of SPINT1-AS1 was significantly and negatively correlated with miR-214 in cervical cancer tissues. Functionally, SPINT1-AS1 expression drove cervical cancer cellular proliferation, migration, and invasion in vitro, as well as tumorigenesis in vivo [60].

## 4. Modulation of DDR Signaling by lncRNAs

As detailed in the introduction, there are two distinct phases of HPV genome replication. During maintenance replication, HPV genomes are replicated in the basal epithelial cells in synchrony with host cellular DNA synthesis. This serves to counteract the dilution of viral episomes after each cell division. Vegetative amplification is a differentiation-dependent process through which genomes are generated at a very high copy number at the sites of double-strand DNA breaks, and viral progeny is produced in terminally differentiated cells [4,5].

**Figure 3:**
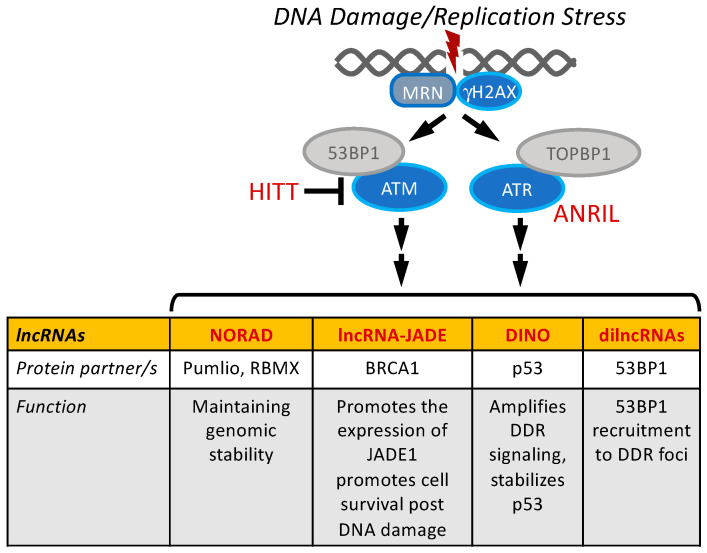
LncRNAs important in DNA damage signaling. LncRNAs present in the box are induced by DNA damage. HITT and ANRIL interact with the DNA damage components ATM and ATR. See text for details and references.

Interestingly, however, DDR signaling is required during both phases of viral genome replication. Ataxia–telangiectasia-mutated (ATM) and ataxia–telangiectasia and Rad3-related protein (ATR) are serine/threonine protein kinases that function as sensors and the key regulators of DDR signaling (Figure 3). ATM is recruited and activated by DNA double-strand breaks and phosphorylates and activates the CHEK2 kinase, whereas ATR is the main kinase that senses DNA damage during the S phase, and it phosphorylates and activates the CHEK1 kinase. ATM activity is needed for vegetative amplification but not for maintenance replication [4], whereas ATR activity is important for maintenance replication [14] and plays a role in vegetative amplification as well [14,61]. During vegetative amplification, viral replication occurs at discrete nuclear compartments termed replication foci, where ATM pathway members colocalize too [5]. The expression of the E7 protein causes increased expression of many components of the ATM signaling pathway including RAD51 and BRCA1 [4]. RAD51 binds the viral genomes, and these components are necessary for productive replication but not for maintenance replication [62]. A more detailed review of the role of DDR during the HPV lifecycle can be found in [63,64].

Not surprisingly, it has been reported that double-stranded DNA breaks result in the differential expression of lncRNAs [65,66] (Figure 3). LncRNA-JADE is transcriptionally activated in response to DNA damage through the ATM pathway [65]. LncRNA-JADE interacts with the BRCA1 protein to promote the Jade family PHD finger 1 (JADE1) transcription. JADE1 encodes a scaffolding protein of human acetylase binding to ORC1 (HBO1) complexes that are involved in histone H4 acetylation [67,68,69,70], thereby regulating gene expression and replication [69,70]. JADE1 can also negatively regulate WNT/b-catenin signaling [71]. LncRNA-JADE is highly expressed in some breast cancers, and the depletion of lncRNA-JADE can sensitize cells to DNA-damaging agents [65].

BRCA1 has also been revealed to interact with other lncRNAs such as the telomeric-repeat-containing RNA (TERRA) to suppress telomere-centered genome instability [72] and damaged-induced lncRNAs (dilncRNAs), which are transcribed from the sites of double-strand DNA breaks [73]. BRCA1 is a component of the HPV replication foci [5] and is necessary for vegetative amplification [62].

The expression of the E2F1-regulated inhibitor of cell death (ERIC; ERICD; XLOC 006942) lncRNA fluctuates during the cell division cycle in concert with E2F1 transcription factor activity. ERIC lncRNA levels increase following treatment with the DNA-damaging agent etoposide, and there is increased etoposide-induced apoptosis in cells where ERIC expression is ablated. Hence, the ERIC lncRNA may serve as a component of a feedback mechanism that controls the cell death response in response to aberrant E2F1 activity [74].

The noncoding RNA activated by DNA damage (NORAD) is a cytoplasmic lncRNA that is activated by DNA damage. It contributes to the maintenance of genomic stability by binding and sequestering members of the Pumilio family of RNA-binding proteins [75,76]. The sequestration of Pumilio proteins by NORAD is necessary for normal mitosis, and NORAD inactivation triggers aneuploidy presumably as a consequence of Pumilio proteins that repress the stability and translation of mRNAs that encode proteins critical for mitosis. NORAD also contributes to the maintenance of genomic stability by interacting with the heterogeneous nuclear ribonucleoprotein G to scaffold a ribonucleoprotein complex that contains TOP1, ALYREF, and the PRPF19–CDC5L complex, which is important for chromosome segregation, replication fork velocity, and cell cycle progression [77].

The lncRNA in nonhomologous end joining (NHEJ) pathway 1 (LINP1) was found to associate with Ku80 and DNA-PKcs in HPV18-positive Hela S3 cervical cancer cells and localizes to the nucleus upon irradiation. The ablation of LINP1 expression resulted in increased levels of cleaved caspase3 and PARP, which enhances cell death upon irradiation, indicating that LINP1 may be important in the repair process of DSBs [78].

The nuclear paraspeckle assembly transcript 1 (NEAT1) has been implicated in regulating the expression of the genes involved in DNA repair processes, including the homologous recombination pathway, and silencing NEAT1 was shown to increase the sensitivity to a variety of DNA damage-inducing chemotherapy agents [79]. NEAT is discussed in more detail in Section 5.

The lncRNA ANRIL (also known as CDKN2B-AS1) is encoded in the antisense orientation of the INK4B/INK4A/ARF locus. A large number of ANRIL splice variants exist, and ANRIL may be a component of a polycomb repressive complex that silences the expression of the INK4B/INK4A/ARF locus in proliferating cells [80]. ANRIL also enhances DNA repair by homologous recombination by binding the ATR protein, thereby protecting it from ubiquitin-mediated proteasomal degradation [81].

The HIF-1alpha inhibitor at translation level (HITT; aka. LINC00637) is a lncRNA that can sensitize cells to apoptosis in response to treatment with DNA damage-inducing compounds. HITT expression is upregulated through a p53-independent mechanism in response to DNA damage. HITT then binds ATM, thereby interfering with the recruitment of ATM to the sites of double-strand DNA breaks by the MRE11/RAD50/NBS1 (MRN) complex [82].

Lastly, our work with the damage-induced noncoding RNA (DINO; DINOL) [83,84] has revealed that DINO can activate the hallmarks of double-strand DNA breaks and ATM activation, although the mechanism is currently unknown [83]. DINO is discussed in greater detail in Section 5.

The research conducted on a group of lncRNAs termed damage-induced lncRNAs (dilncRNAs) might be of particular interest to those who study the architecture of HPV replication foci. As mentioned above, dilncRNAs are synthesized by RNA polymerase II after MRN complex recruitment to the sites of double-strand DNA breaks. The dilncRNAs are then processed by DICER and DROSHA into smaller noncoding RNAs, referred to as DNA damage response RNAs (DDRNAs), which then bind the previous dilncRNA strands. Both the DDRNA and dilncRNA together bind 53BP1 at its Tudor domain at the site of the double-strand DNA break foci and are important for 53BP1 recruitment. This process can be inhibited by using antisense oligonucleotides that disrupt dilncRNA and DDRNA binding, which provides the foundation for a potential therapeutic strategy [85].

These DNA:RNA hybrids have been shown to co-localize to BRCA1 in S-phase cells, and BRCA2 mediates the recruitment of RNAse H2 to induce their degradation at these sites [73]. E7 has been reported to increase the expression of RNAseH2A through an E2F1-dependent mechanism [86]. Hence, it is tempting to speculate that dilncRNAs may be present at HPV replication foci, where they may be targeted with antisense oligonucleotides to prevent viral replication. While it is presently unknown if there are specific sites where viral replication foci form in the nucleus, it has been shown that the late viral replication foci tend to form adjacent to common fragile sites [87]. Recent publications provide evidence that transcription and replication are spatially organized at the viral replication foci, and RNA poll II recruitment occurs at the “satellite” locations of the foci [88].

## 5. LncRNAs That Are Connected to p53 Activity

The p53 tumor suppressor is a transcription factor that functions as a major downstream mediator of DDR signaling. DNA damage causes p53 activation by post-translational modifications including phosphorylation by DDR kinases such as ATM. These post-translational modifications cause p53 stabilization and result in the activation of cytotoxic or cytostatic transcriptional responses. Depending on cell type and/or the extent of DNA damage, p53-induced transcriptional responses can result in cell death, senescence, cell cycle arrest, and cellular differentiation. The p53 tumor suppressor is mutated in the majority of human solid tumors [89]. In contrast, however, HPV-associated cancers retain wild-type p53 [90]; however, p53 activity is inhibited by the high-risk HPV E6 proteins, which target p53 for rapid proteasomal degradation [91]. It has been suggested that the HPV-associated tumors retain some p53 tumor suppressor activity, which may account for the increased sensitivity of HPV-positive cancers compared with their HPV-negative counterparts [92]. The remaining, diminished activity of p53 may be required for the HPV life cycle [93].

It is important to note that p53 activation in keratinocytes can trigger differentiation through the activation of NOTCH1, rather than cell death [94]. There has been evidence that E6 expression is restricted to the basal layers of the epithelium in organotypic raft culture and that the disruption of NOTCH1 by E6 occurs in a p53-dependent manner [95].

**Figure 4:**
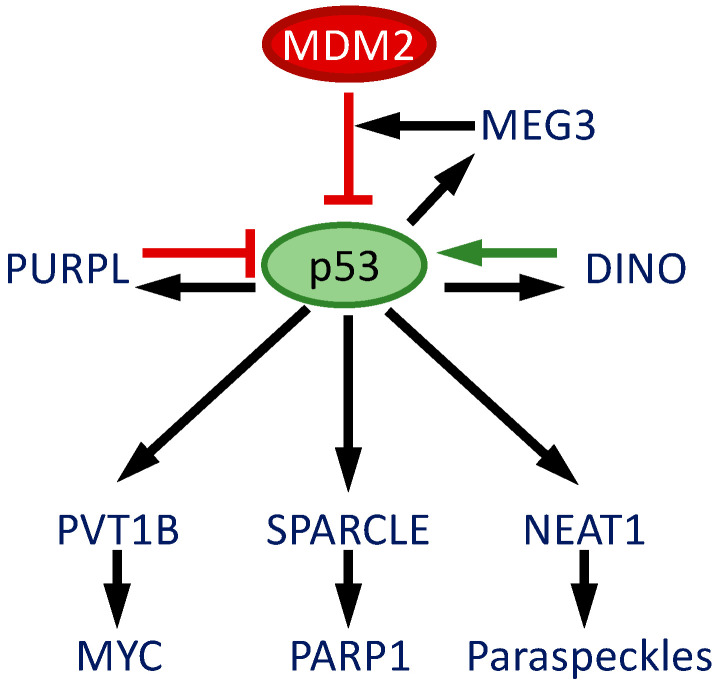
p53-related lncRNAs. p53 induces the expression of multiple lncRNAs that trigger a variety of cellular signaling circuits and biological responses. Some lncRNAs further induce and stabilize p53, while others restrict p53 levels. See text for details and references.

Multi-omic approaches have been crucial in identifying p53 transcriptional targets. The integration of RNA-seq with p53 ChIP-seq analysis of HCT116 cells, treated with a DNA-damage-inducing agent, 5-fluorouracil (5-FU), identified 18 lncRNAs that are p53 transcriptional targets in response to DNA-damage treatment [96]. Some of these p53-responsive lncRNAs have been studied mechanistically, and these may provide attractive therapeutic targets to regulate p53 activity in various cancers, particularly those that still maintain wild-type p53 such as HPV-positive cancers (Figure 4).

NEAT1, a particularly well-studied lncRNA, is a p53 transcriptional target, and its paraspeckle formation following genotoxic stress is p53-dependent [97,98]. Neat1 is not required for p53-induced DNA damage responses in mice [98]. There is still no consensus on whether Neat1 has oncogenic or tumor-suppressive activities. Neat1^−/−^ mice were shown to be resistant to DMBA/TPA-induced skin carcinogenesis [97], whereas in other experiments, Neat1^−/−^ mice were more susceptible to Ras oncogene-driven pancreatic cancer initiation. Mouse embryo fibroblasts (MEFs) derived from Neat1^−/−^ mice showed enhanced transformation in vitro assays [98]. These overtly contradictory results indicate that NEAT1 may have cell-type-specific, p53-dependent oncogenic activities.

The p53 upregulated regulator of p53 levels (PURPL) is a nuclear lncRNA that is important in restraining basal p53 levels. When PURPL is depleted in colorectal cancer cells, basal p53 levels are elevated, which resulted in growth defects of cancer cells in vitro and in mouse xenografts. This is through an association binding with MYBBP1A via the HuR adaptor protein. The HuR protein is known to bind and stabilize p53. The knockdown of MYBBP1A in PURPL-depleted cells causes a decrease in p53 levels and rescues the proliferation defect [99].

The expression of the cytosolic p53-related lncRNA (P53RRA; LINC00472) is downregulated through epigenetic mechanisms in many cancers and has tumor suppressor activities. Low P53RRA expression significantly correlated with poor survival in patients with breast and lung cancers harboring wild-type p53 [100]. Ectopic P53RRA expression causes growth arrest and the apoptosis of cancer cells in vitro in a mouse xenograft model [100]. P53RRA binds Ras GTPase-activating protein-binding protein 1 (G3BP1). This displaces p53 from G3BP1, thereby increasing p53 levels and activity in the nucleus, which causes cell cycle arrest and programmed cell death, including apoptosis and ferroptosis [100].

The maternally expressed gene 3 (MEG3) lncRNA is induced in endothelial cells in response to p53 activation via DNA damage. MEG3 depletion induces DNA damage concurrently activating and amplifying p53 signaling, which results in cell cycle arrest and cell death. MEG3 is thought to function by stabilizing the interaction between p53 and MDM2, thereby accelerating p53 degradation and dampening p53-mediated cytotoxic responses to DNA damage [101].

The suicidal PARP-1 cleavage enhancer (SPARCLE) is a nuclear lncRNA that was identified as a component of the “late” DDR response. SPARCLE levels increase at 24 h after DNA damage through a p53-dependent mechanism. It binds PARP1 and promotes the cleavage of PARP1 via caspase-3. Hence SPARCLE is thought to signal p53-mediated apoptosis after extensive DNA damage that can no longer be successfully repaired. The ectopic expression of the N-terminal of the PARP1 fragment, which is sufficient to inhibit DNA repair, restores the cell death response in SPARCLE-deficient cells [102].

PVT1B, an isoform of the PVT1 lncRNA, is located 50 kb downstream of the *MYC* oncogene and has been identified as a p53-responsive transcript. PVT1B suppresses MYC transcription in *cis*, thereby inhibiting MYC-dependent cellular proliferation and tumor growth but not malignant progression in a mouse model of lung carcinogenesis. Hence, PVT1B is an important component of p53-dependent cytostatic transcriptional processes by shutting down oncogenic MYC signaling in response to p53 activation [103].

The RNA component of mitochondrial RNA-processing endoribonuclease (RMRP) is a lncRNA that inhibits p53 activity by retaining the SNRPA1 protein in the nucleus, where it promotes MDM2-mediated p53 ubiquitination and degradation. RMRP inhibition causes p53 activation, which enhances the sensitivity of colorectal cancer cells to PARP inhibition. Interestingly, RMRP expression increases in response to PARP inhibitors [104].

The lncRNA DINO has a well-established role in DNA damage signaling and functions as a p53-dependent enhancer of cell death in response to doxorubicin and other DNA-damaging agents [83,84]. DINO was shown to activate, stabilize, and amplify p53 signaling [84]. DINO expression is low in HPV-positive cervical cancer lines, consistent with the functional inactivation of p53 by E6/E6AP-mediated ubiquitination and proteasomal degradation. Ectopic DINO expression in HPV-positive cervical cancer cell lines was shown to reactivate p53, as manifested by a significant sensitization to DNA damage-inducing chemotherapy agents. Unexpectedly, DINO expression was also induced in a p53 mutant cervical cancer cell line in response to DNA damage [83]. Furthermore, there was evidence that the induction of DINO expression in response to DNA damage may be, at least in part, independent of the ATM/CHK2/p53 axis. DINO expression is silenced by DNA methylation in human cancers that retain wild-type p53, suggesting a model whereby the silencing of DINO expression represents an alternative mechanism that can lead to the loss of p53 tumor suppressor activity [105]. DINO^−/−^ mice spontaneously developed soft tissue and osteosarcomas, indicating that the genetic loss of DINO is sufficient to trigger tumorigenesis. This supports the notion that DINO has tissue-specific tumor suppressor activities. Interestingly, soft-tissue sarcomas and osteosarcomas are among those human tumor types that often retain wild-type p53, while DINO expression is epigenetically silenced. Lastly, because DINO and the canonical p53-responsive p21^CIP1^ (CDKN1A) protein are transcribed from the same locus (albeit in different orientations and from different promoters), it may be argued that p21^CIP1^ and DINO may have overlapping tumor-suppressive activities. This is unlikely to be the case, since in contrast to the DINO^−/−^ mice, the p21^CIP1−/−^ mice do not spontaneously develop tumors [106]. Overall, these important findings established DINO and not p21^CIP1^ as an important mediator of the tumor-suppressive activities of p53 [107].

The p53 tumor suppressor, the “guardian of the genome” [108], has been widely studied in the past 40 years, and significant advancements in our understanding of this protein have been achieved [109,110]. However, the complete functional understanding of the mechanisms and consequences of p53 expression cannot be fully elucidated without a detailed and mechanistic understanding of the contributions of noncoding RNAs. In the context of cancers, whether HPV-associated or not, restoring wild-type p53 function in tumors has been an attractive option for the design of future therapies. However, such therapies will need to be tailored to the genetics of the specific tumor type. Given that DINO is an important mediator of p53 tumor suppressor activity, it is a promising target to restore this pathway downstream of p53.

## 6. Conclusions

The maintenance of the basal cell identity of infected cells, the subversion of epithelial cell differentiation, and the modulation of DDR signaling are crucial to the HPV lifecycle. Important insights have been gleaned by identifying and investigating the protein regulators of these pathways. While these studies are still in their infancy, it is obvious that lncRNAs play well-defined, important mechanistic roles in these pathways and serve as targets by HPV proteins and may contribute to the oncogenicity of the cancer-associated high-risk HPVs. The HPV E6 and E7 proteins are the main drivers of HPV carcinogenesis, and their expressions cause profound alteration in the lncRNA transcriptome. Mechanistic studies on lncRNAs are inherently complicated by our current lack of predictive algorithms that could guide mechanistic studies. Similar tools are available and greatly simplify studies of protein targets. Despite these challenges, researchers should not shy away from such studies. They promise to provide exciting insights and lead to the discovery of novel regulatory nodes that are targeted by HPVs to establish long-term persistent infections and complete their productive lifecycle and, in the case of high-risk HPVs, may trigger carcinogenesis. In addition, lncRNAs provide attractive targets for the treatment of HPV-associated cancers or as antivirals to extinguish persistent HPV infections.
